# Male Sex, B Symptoms, Bone Marrow Involvement, and Genetic Alterations as Predictive Factors in Diffuse Large B-Cell Lymphoma

**DOI:** 10.3390/ijms26115087

**Published:** 2025-05-26

**Authors:** Matej Panjan, Vita Šetrajčič Dragoš, Gorana Gašljević, Srdjan Novaković, Barbara Jezeršek Novaković

**Affiliations:** 1 Division of Medical Oncology, Institute of Oncology Ljubljana, 1000 Ljubljana, Slovenia; 2 Faculty of Medicine, University of Ljubljana, 1000 Ljubljana, Slovenia; vsetrajcic@onko-i.si (V.Š.D.); srdjan@mankuc.com (S.N.); 3 Department of Molecular Diagnostics, Institute of Oncology Ljubljana, 1000 Ljubljana, Slovenia; 4 Department of Pathology, Institute of Oncology Ljubljana, 1000 Ljubljana, Slovenia; ggasljevic@onko-i.si; 5 Faculty of Medicine, University of Maribor, 2000 Maribor, Slovenia

**Keywords:** DLBCL, genetic classification, predictive, B symptoms, sex, bone marrow involvement

## Abstract

Approximately 40% of patients with diffuse large B-cell lymphoma (DLBCL) are not cured with first-line chemoimmunotherapy, resulting in poor prognosis. Schmitz et al. classified DLBCL into four prognostic genetic groups using whole-exome sequencing. We applied a simplified approach using a targeted next-generation sequencing assay (Archer FusionPlex Lymphoma Assay) to analyze samples from 105 patients—53 with a progression-free survival (PFS) < 2 years (the “Relapse group”) and 52 with a PFS > 5 years (the “Remission group”) following first-line systemic treatment. Patients were classified according to Schmitz et al. into the following categories: “MCD” (*MYD88^L265P^* and *CD79B* alteration), “N1” (*NOTCH1* alteration), “BN2” (*NOTCH2* alteration and *BCL6* translocation), and “EZB” (*EZH2* alteration and *BCL2* translocation). The predictive value of this simplified genetic classification and of relevant clinical features were evaluated. The “Relapse group” included more patients classified as MCD and N1, while fewer were classified as EZB and BN2. Also, cell-of-origin (COO) characteristics and the size of N1 aligned with the classification of Schmitz et al. However, the limited sample size precludes definitive conclusions about the predictive value of our simplified approach. Additionally, male sex, B symptoms, and bone marrow involvement were associated with relapse. Therefore, these clinical features may be useful in predicting outcomes until an effective molecular classification is widely adopted.

## 1. Introduction

Diffuse large B-cell lymphoma (DLBCL) is a clinically and molecularly heterogeneous type of non-Hodgkin lymphoma—the most common subtype—and has an annual incidence of about 5 per 100,000 individuals [1,2]. Approximately 40% of patients are not cured with first-line R-CHOP (rituximab, cyclophosphamide, doxorubicin, vincristine, and prednisolone); additionally, 10% fail to achieve remission and 20–30% relapse from complete remission, both of which are associated with an adverse prognosis [3,4]. Most DLBCL relapses occur within two years after first treatment and early relapses are likely genetically distinct and prognostically inferior compared to later relapses [5,6].

Prognostic and predictive factors help to identify patients at a higher risk of progression, who may require closer follow-up or alternative therapies as they become available [7].

The International Prognostic Index (IPI) is a widely recognized prognostic clinical tool comprising age, stage, involvement of more than one extranodal site, the World Health Organization (WHO) performance status (PS), and increased value of lactate dehydrogenase (LDH) [8,9]. Additional markers of poor prognosis include *MYC* and *BCL2* overexpression, the translocation of *MYC* and *BCL2* and/or *BCL6*, and bone marrow involvement [10,11].

The cell-of-origin (COO) classification of DLBCL plays a fundamental role in understanding the prognostic heterogeneity of the disease. Based on gene expression profiling (GEP), it distinguishes between activated B-cell (ABC) and germinal-center B-cell (GCB) subtypes, with the ABC subtype typically displaying a worse outcome [12]. However, as the COO classification reflects the phenotypic rather than the genetic causes of malignant transformation, it offers limited therapeutic guidance. It also leaves 10–15% of cases unclassified and is not easily adopted in clinical settings. Therefore, immunohistochemical methods, such as the Hans algorithm, are used as prognostic surrogates [13].

Next-generation sequencing (NGS) enables a detailed analysis of DLBCL’s genetic diversity. It identifies both driver and secondary mutations and can group tumors by shared genetic profiles. These profiles, despite being complex, are often defined by a few key mutations with strong pathological relevance, providing insights into how mutational patterns drive transformation [14,15,16].

Schmitz et al. conducted one of the first studies establishing genetically and prognostically distinct DLBCL groups. Using the whole-exome sequencing (WES) of 574 cases, they identified four genetic groups based on 79 genes [14]. Each group was characterized by one or two commonly affected genes—“MCD” was characterized by *MYD88^L265P^* and alteration in *CD79B*; “N1” was characterized by *NOTCH1* aberration’ “BN2” was characterized by altered *NOTCH2* and *BCL6* translocation; and “EZB” was characterized by *EZH2* aberration and *BCL2* translocation. MCD and N1 corresponded to the ABC, the EZB to GCB, and the BN2 to mixed subtypes. EZB and BN2 were associated with a better progression-free (PFS) and overall survival (OS), while MCD and N1 displayed poorer outcomes. Still, 55% of cases remained unclassified as no prominent genetic features were found [14]. This classification was later reevaluated by Wright et al. using a new algorithm. Among the previously unclassified cases, groups “A53”, with *TP53* mutation and aneuploidy, and “ST2”, with *SGK1* and *TET2* mutations, were defined, thereby enabling a classification of up to 63% of patients [17].

In a similar genetic classification attempt by Chapuy et al., WES was performed on 304 DLBCL cases. A total of 98 cancer driver genes were identified, with a median of 17 genetic alterations per tumor. The study defined five genetic groups. “C1” was characterized by *NOTCH2* mutation and *BCL6* translocation, “C2” was characterized by *TP53* inactivation, “C3” was characterized by *BCL2* translocation and *EZH2* mutation, “C4” was characterized by the mutation of histone genes, and “C5” was characterized by 18q gain, as well as *MYD88^L265P^* and *CD79B* mutation [15]. Three of these groups were similar to those found in Schmitz’s study. Namely, C1 resembled BN2, C3 resembled EZB, and C5 was similar to MCD [14,15]. C5 and C1 were predominantly of the ABC subtype. C3 and C4 were of the GCB subtype, whereas C2 was of a mixed subtype. In Chapuys’ study, only 4% of cases, labeled as “C0”, remained unclassified. The outcomes were shown to be favorable in C0, C1, and C4, while adverse outcomes were observed in C3 and C5 [15].

Lacy et al. analyzed 839 DLBCL cases using a panel of 293 genes associated with hematologic neoplasms. They identified 117 alterations occurring in at least 1% of cases, with a median of seven mutations per patient. The five recognized genetic groups were “MYD88”, “BCL2”, “TET2/SGK1”, “SOCS1/SGK1”, and “NOTCH2”, with 27% of cases remaining genetically unclassified. “MYD88” commonly included *MYD88*^L265P^ and *CD79B* mutations, “BCL2” included *BCL2* translocations and *EZH2* mutations, and “NOTCH2” contained *NOTCH2* mutations. Based on GEP COO, “MYD88” and “BCL2” were mostly of the ABC subtype, whereas “NOTCH2” was of a mixed subtype [16]. Again, these three groups displayed similarities with the MCD, EZB, and BN2 subtypes described in Schmitz’s work, as well as to C5, C3, and C1 described in Chapuy’s work [14,15].

Reddy et al. conducted a large genetic study of 1001 DLBCL cases and identified 150 driver genes with a mean of 7.75 mutations per tumor. The most frequently altered genes were *KMT2D*, *BCL2*, and *MYD88*, with *NOTCH2*, *EZH2*, and *CD79B* being among the most commonly mutated genes. Of note, the *EZH2* alteration was associated with the GCB subtype, whereas the *MYD88* and *CD79B* alterations were more common in the ABC subtype. The latter aberration was also associated with a negative outcome. However, this study did not aim to group its data into comprehensive genetic groups [18].

In our study, DLBCL cases were classified into four genetic groups based on the classification system proposed by Schmitz et al., using alterations in seven genes that are commonly affected in DLBCL. We tested the following three hypotheses that refer to patient characteristics at diagnosis and were defined prior to all analyses:

1. The “Relapse” group has a higher proportion of the MCD and N1 subtype, as well as a lower proportion of the BN2 and EZB genetic groups, compared to the “Remission” group.
2. The “Relapse” group shows a higher proliferation index and a greater proportion of ABC subtype cases than the “Remission” group.
3. Patients in the “Relapse” group have higher IPI scores, as well as a greater frequency of B symptoms and bone marrow involvement, compared to the “Remission” group.

In our study, we aimed to assess the predictive value of a simplified, RNA-based genetic classification and of the relevant clinical characteristics of DLBCL patients.

## 2. Results

Out of the 105 patients with DLBCL included in our study, 52 were in the “Remission” group and 53 were in the “Relapse” group. In five cases, genetic analysis and GEP classification according to the COO were unsuccessful (QC failed), and they were excluded from analyses that included these variables. The patient characteristics are presented in Table 1.

**Figure 1:**
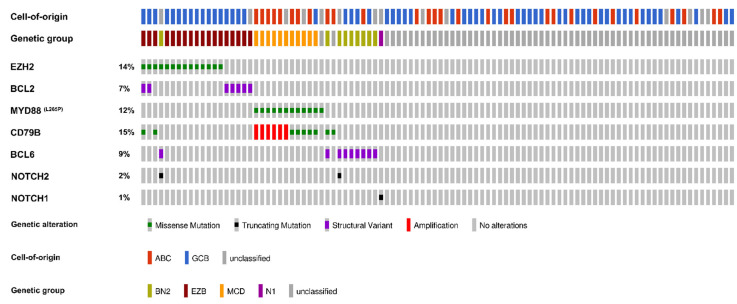
A schematic presentation of the cell-of-origin classification determined by gene expression profiling and genetic classification based on alterations in *EZH2*, *BCL2*, *MYD88*, *CD79B*, *BCL6*, *NOTCH2*, and *NOTCH1* of diffuse large B-cell lymphoma cases. Cases with alterations in *CD79B* and *MYD88* were classified as MCD; cases with alterations in *EZH2* and/or *BCL2* were classified as EZB; cases with alterations in *BCL6* and/or *NOTCH2* were classified as BN2; and cases with alterations in *NOTCH1* were classified as N1. The remaining cases were genetically unclassified. ABC—activated B-cell; GCB—germinal center B-cell. N = 100.

Figure 1 depicts the COO classification determined by the GEP and genetic classification of DLBCL samples into groups EZB, MCD, BN2, and N1 based on alterations in *EZH2*, *BCL2*, *MYD88*, *CD79B*, *BCL6*, *NOTCH2*, and *NOTCH1*. *BCL2* and *BCL6* were uniformly afflicted by a translocation to the *IgH* gene, *CD79B* was afflicted by a mutation or amplification, whereas *EZH2*, *MYD88*, *NOTCH2*, and *NOTCH1* were afflicted by mutations. The exact list of alterations used for classification is given in Supplementary Table S1.

The EZB group had the most numerous cases, with 18, followed by 11 cases for MCD, 9 for BN2, and 1 N1 case. Sixty-one patients (61%) remained genetically unclassified as none of the specific alterations were found; however, in five cases, genetic analysis was unsuccessful. The majority of the MCD and the only N1 case were in the “Relapse” group, whereas more than half of the EZB and BN2 groups were in the “Remission” group (Table 2).

**Figure 2:**
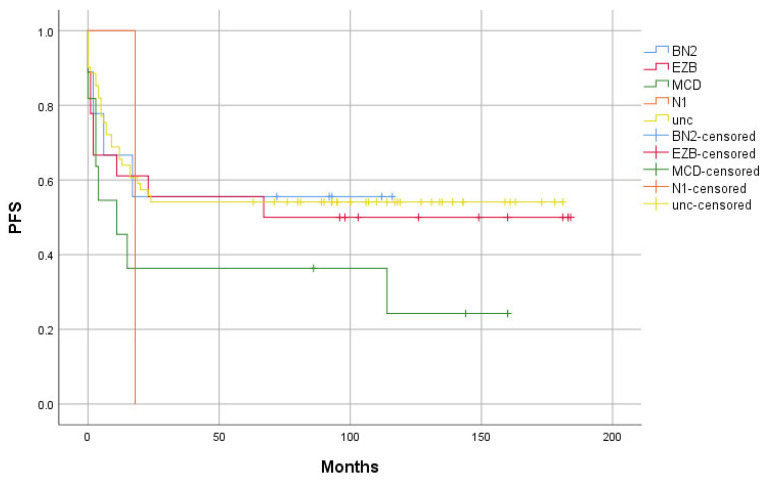
Progression-free survival (PFS) of diffuse large B-cell lymphoma cases classified into BN2, EZB, MCD, and N1 genetic groups, as well as of genetically unclassified cases, after the first systemic treatment. unc—unclassified. N = 100.

The difference in proportions of the EZB, MCD, BN2, and N1 genetic groups between the “Relapse” and “Remission” groups did not reach statistical significance, neither individually nor in the logistic regression model. The PFS of the genetic groups is shown in Figure 2.

**Figure 3:**
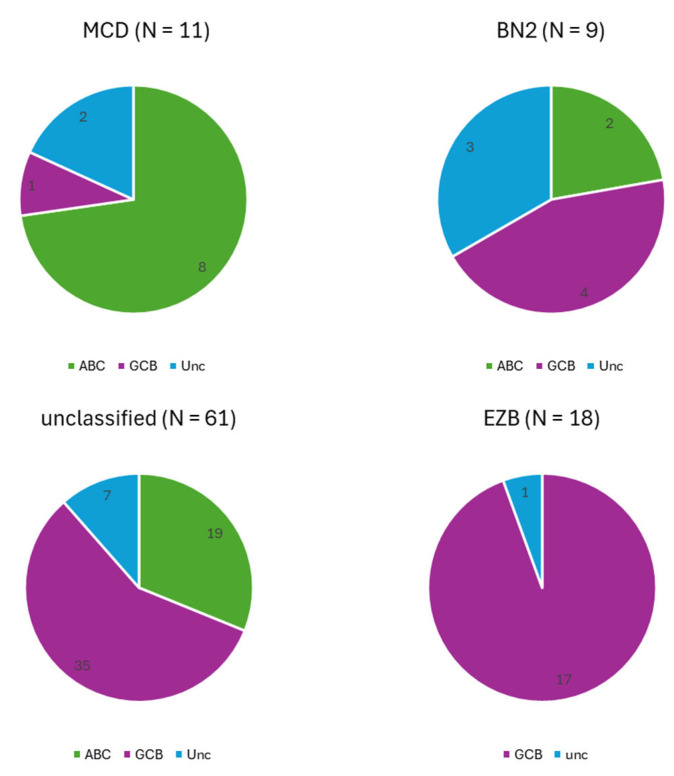
The cell-of-origin characterization determined by gene expression profiling of diffuse large B-cell lymphoma cases, classified into MCD, BN2, and EZB genetic groups, and of unclassified cases. The only N1 case remained unclassified. ABC—activated B-cell; GCB—germinal center B-cell; unc—unclassified.

The COO characterization performed by the GEP of the genetic groups is shown in Figure 3. The MCD cases were predominantly of the ABC subtype, whereas the EZB cases were almost exclusively of the GCB subtype. The N1 case remained unclassified according to GEP, but wasclassified as the ABC subtype according to the Hans algorithm. The BN2 cases included balanced proportions of the ABC and GCB subtypes, as well as unclassified cases, whereas more than half of the genetically unclassified cases were of the GCB subtype (Figure 3).

According to the COO classification with GEP, there were 14 unclassified cases. Overall, the ratio of ABC/GCB cases was 29/57. In the “Relapse” group, there were 35% ABC and 48% GCB cases, whereas in the “Remission” group, we identified 23% ABC and 65% GCB cases. The proportion of ABC cases was thus higher in the “Relapse” compared to the “Remission” group, but the difference was not statistically significant (*p* = 0.174).

The Hans algorithm and COO determined by GEP matched in 76% of cases. The proportion of the ABC subtype according to the Hans algorithm in the “Relapse” group was 44% and thus higher compared to that of the “Remission” group, where it was 35%; however, the difference was not significant.

The main clinical characteristics of the genetic groups are presented in Table 3. BN2 was the oldest and N1 was the youngest group. Tested alongside other genetic groups, EZB was significantly associated with an increased LDH (*p* = 0.012), whereas none of the genetic groups were significantly associated with stage or a WHO PS > 1. Bone marrow involvement was less common in the EZB group, but no significant association was present (Table 3). In the whole cohort, elevated LDH was associated with a higher stage (*p* < 0.001). With FISH analysis, we identified three cases of DLBCL/high-grade B-cell lymphoma (HGBCL) with *MYC* and *BCL2* rearrangements. Two of them were found in the EZB group and one was found in the unclassified group.

In 30 cases, evidence of indolent lymphoma was present at or before the diagnosis of DLBCL, suggesting that the disease likely evolved from an indolent precursor. None of these patients received systemic therapy for indolent lymphoma prior to DLBCL treatment. Transformation occurred from follicular lymphoma (FL) in 10 cases, from marginal zone lymphoma (MZL) in 6 cases, and from chronic lymphocytic leukemia (CLL; “Richter transformation”) in 3 cases. In 11 cases, the type of indolent lymphoma could not be determined. There were 20 transformed cases in the “Relapse” group and 10 in the “Remission” group, with transformation being significantly associated with relapse within two years after treatment (*p* = 0.036). All three Richter transformation cases were found in the “Relapse” group. Among the 10 cases transformed from FL, two were classified as EZB—one with an *EZH2* mutation and one with a *BCL2* translocation. Seven FL-transformed cases were unclassified, and one had an unsuccessful genetic analysis. Of the six MZL-derived cases, one was classified as MCD, one was classified as BN2, and the remaining four were unclassified. Of the three Richter transformation cases, one was categorized as N1, while two remained unclassified (Table 3). The clinical characteristics of the “Relapse” and “Remission” groups are presented in Table 4 and Table 5. The stage was determined primarily with PET-CT scan (Positron Emission Tomography–Computed Tomography) and bone marrow biopsy. Of the five IPI criteria, LDH was elevated significantly more often and the stage was significantly higher in the “Relapse” group compared to the “Remission” group. There was no significant difference in the remaining three criteria (Table 4 and Table 5). In a multivariate analysis of the IPI criteria, stage and elevated LDH were associated with relapse (*p* = 0.004 and 0.021, respectively). IPI score was significantly higher in the “Relapse” group compared to the “Remission” group (*p* = 0.001).

An evaluation of the clinical characteristics not included in the IPI showed no significant difference in median MIB-1 proliferation index between the two groups. B symptoms were initially associated with relapse within two years after treatment (*p* = 0.004), but this association lost significance after adjusting for IPI (*p* = 0.164). Patients with B symptoms had a significantly higher disease stage compared to those without (*p* < 0.001). Male sex was also associated with relapse (*p* = 0.039), and this association remained significant after controlling for IPI (*p* = 0.032).

Lymphoma bone marrow involvement was classified as “concordant” if only DLBCL involvement was present; in all other cases, it was classified as “discordant”. There were 6 concordant and 26 discordant cases. Bone marrow involvement was significantly associated with a relapse within two years after treatment (*p* < 0.001), and this association remained significant after adjusting for IPI (*p* = 0.010). Both concordant and discordant involvement types were individually associated with a relapse (*p* = 0.027 and *p* = 0.007, respectively). No correlation was observed between bone marrow involvement and elevated LDH levels.

## 3. Discussion

In the study by Schmitz et al., which employed WES, 79 altered genes contributed to the definition of four genetic DLBCL groups. However, each group was primarily characterized by one or two frequently altered genes. In the MCD group, 72% of patients presented with *MYD88^L265P^* and 52% presented with *CD79B* alterations; in the BN2 group, 74% presented with a *BCL6* translocation and 44% presented with a *NOTCH2* mutation. In the N1 group, all cases had a *NOTCH1* mutation, while in the EZB group, 68% showed a *BCL2* translocation and 44% showed an *EZH2* mutation [14]. These genetic alterations represent early, clonal events in lymphoma development [15]. Unlike Schmitz et al., we based our condensed classification on these seven genes alone using RNA sequencing, aiming to test its predictive value alongside clinical characteristics.

Alterations in *CD79B* and *MYD88* suggest the dysregulation of the B-cell receptor and Toll-like receptor pathways, both activating NF-κB and promoting oncogenesis [14,15]. In the C5 group, and likely the MCD group, the predominant mutational process is driven by activation-induced cytidine deaminase (AID) and somatic hypermutation [15]. These alterations are also common in extranodal DLBCL, such as in primary central nervous system (CNS) and testicular types [19]. Schmitz et al. observed a trend of increased extranodal involvement in the MCD group, while Chapuy et al. reported a higher proportion of primary testicular and CNS lymphomas in the C5 group [14,15]. In our study, the MCD group was not associated with the more frequent involvement of multiple extranodal sites. Moreover, conclusions regarding testicular and CNS lymphomas could not be drawn, as these cases were excluded from our study.

There was some evidence of an inferior outcome in the MCD group as most of the included cases experienced a relapse within two years after treatment. Considering the two MCD-defining mutations individually, the majority of patients with *MYD88^L265P^* and *CD79B* alterations relapsed. *MYD88^L265P^* is the most common *MYD88* variant, and its negative prognostic impact may be specific to this particular mutation [20]. It is also recognized as an independent adverse prognostic marker in DLBCL [20,21,22]. Likewise, the second MCD-defining alteration—*CD79B*—has been linked to poorer prognosis [18,22], although evidence supporting its standalone significance is less robust. Both the MCD group and the individual *MYD88^L265P^* and *CD79B* alterations were more common in ABC-subtype DLBCL, which is consistent with the existing literature [14,18,22].

A narrow majority of BN2 cases did not experience a relapse within two years; this group was indeed prognostically superior in Schmitz’s study. The molecular characteristics of BN2, such as the *NOTCH2* mutation and the *BCL6* translocation, are shared by marginal zone lymphoma (MZL) and transformed MZL [23,24]. This may indicate that the BN2 group partly shares its pathogenesis with MZL, or that transformation occurred from an occult MZL [14]. However, our BN2 group did not show a higher rate of transformed MZL, which is consistent with Chapuy’s C1 group, where no transformed MZL cases were found [15].

The N1 group was the smallest genetic subgroup in both our study and Schmitz’s, and was the only one defined by a single alteration—*NOTCH1*—which was present in all N1 patients [14]. Our single N1 case had a poor outcome in terms of both PFS and OS, echoing findings from Schmitz and others linking *NOTCH1* mutation with worse prognosis [14,16,17,25]. Some studies have not identified the N1 group or an equivalent group, which is likely due to the low prevalence of mutation in DLBCL [15,16]. The *NOTCH1* alteration is also frequently seen in CLL [26], and our N1 patient indeed had experienced Richter transformation.

The majority of our EZB cases did not relapse within two years, which is consistent with Schmitz’s report of a favorable prognosis for this group [14]. The EZB group and Chapuy’s C3 group are genetically GCB-predominant profiles, characterized by genetic alterations with epigenetic influence and *BCL2* translocation [14,15]. *EZH2* mutations and *BCL2* translocations are characteristic of both follicular lymphoma (FL) and DLBCL, which has transformed from FL [26,27]. However, in our cohort, only two transformed FL cases were found in the EZB group, and neither had an *EZH2* alteration or a *BCL2* translocation.

In the “Relapse” group, we observed a higher proportion of the MCD and N1 subtypes, as well as a lower proportion of the BN2 and EZB genetic groups compared to the “Remission” group. Therefore, we can confirm the first hypothesis.

Comparing the clinical characteristics between the genetic groups, the EZB group had the highest proportion of cases with elevated LDH, a finding that was not reported elsewhere [14,15,16]. The disease stage was not higher in the EZB group compared to the other groups. Unlike Schmitz’s study, our EZB cases did not show a better WHO performance status [14]. Notably, two of the three DLBCL/HGBCL cases with both *MYC* and *BCL2* rearrangements were in the EZB group. Similarly, in the studies by Chapuy and Lacy, the cases with *MYC* and *BCL2* translocations were found to be more frequent in the C3 and BCL2 subgroups [15,16]. Despite the higher incidence of these cases, which are typically associated with a poorer prognosis, neither the BCL2 group, C3 group, or our EZB subgroup demonstrated an inferior outcome.

Targeted therapies based on genetic subtypes have shown encouraging outcomes in DLBCL. Zhang et al. classified patients using a genetic stratification similar to Schmitz’s and Chapuy’s models, and customized treatment by adding targeted agents to R-CHOP. Specifically, ibrutinib was used for MCD- and BN2-like subtypes, lenalidomide was used for N1-like subtypes, and the histone deacetylase inhibitor tucidinostat was used for EZB-like groups. These genetically tailored approaches yielded higher complete response rates than standard R-CHOP alone [7]. Notably, ibrutinib was particularly effective in DLBCL with *MYD88^L265P^* and *CD79B* alterations, highlighting the therapeutic value of molecular subtyping [28].

The GEP COO classification of our genetic subgroups closely mirrored earlier findings. The MCD group aligned with the ABC subtype, which was consistent with the findings of Schmitz’s study. Likewise, Chapuy’s C5 group and Lacy’s MYD88 group also corresponded to the ABC subtype. The N1 case remained unclassified according to GEP. In Schmitz’s study, the expression profile of this group aligned with the ABC subtype, though less distinctly than MCD, which may explain our findings. The EZB cases in both our and Schmitz’s cohorts were mainly of the GCB subtype, which is similar to Chapuy’s C3 group and Lacy’s BCL2 group. The BN2 group showed a mixed profile, as did Schmitz’s BN2 and Lacy’s NOTCH2 groups, while Chapuy’s C1 group mostly comprised the ABC subtype [14,15,16].

The ABC subtype frequency was higher in the “Relapse” group, both according to GEP and the Hans algorithm. While GEP COO’s prognostic value is well established, a large meta-analysis questioned the value of the Hans algorithm [29,30]. Our results seem to confirm the utility of GEP COO but also imply the clinical utility of the Hans algorithm. No conclusion regarding the superiority of any of these methods can be drawn from our data.

The MIB-1 index, reflecting tumor proliferation, showed no difference between the “Relapse” and “Remission” groups—mirroring the findings from a study of over 900 DLBCL cases that found no association with PFS or OS [31]. Despite more ABC cases in the “Relapse” group, the proliferation rates did not differ significantly, leading us to reject the second hypothesis.

In our study, IPI was clearly associated with a relapse within two years after treatment. This supports its known prognostic role, as demonstrated by Ziepert et al. [8,9]. Notably, in our study, among IPI’s five criteria, only disease stage and elevated LDH were significantly associated with a relapse.

In our series, both concordant and discordant bone marrow involvement were associated with a relapse within two years after treatment. Concordant involvement, in particular, has been linked to poorer outcomes in other studies [11,32,33]. However, bone marrow involvement was not associated with elevated LDH, as reported elsewhere [11,32]. As an extranodal site, it is indirectly considered in the IPI, yet its association with a relapse remained significant even when controlled for IPI.

As hypothesized, B symptoms were significantly associated with a relapse within two years after treatment. These symptoms result from systemic inflammation, with elevated C-reactive protein (CRP) and interleukin-6 (IL-6), which are both linked to poor outcomes [34,35]. Notably, patients with B symptoms had higher disease stages. Since the stage was one of the two IPI components that were significantly associated with a relapse, it is understandable that the association between B symptoms and relapse diminished when adjusted for the IPI.

Given that patients in the “Relapse” group exhibited higher IPI scores and a greater incidence of B symptoms and bone marrow involvement compared to those in the “Remission” group, these findings support our third hypothesis.

In our study, there was a significant association between male sex and relapse, a factor which is not included in the IPI. This association persisted after adjustment for IPI and has been supported by prior studies. Namely, a meta-analysis of 5635 DLBCL patients demonstrated an inferior OS in male patients after the first standard systemic treatment [36]. One explanation for this observation is the faster rituximab clearance in males, possibly reducing treatment efficacy, suggesting that higher rituximab doses for male patients may be beneficial [37,38].

In our study, transformations from indolent lymphoma were also associated with a relapse within two years after treatment. While poorer outcomes have been reported for transformed cases compared to de novo DLBCL, Richter transformation, in particular, is related to worse prognosis, and all such cases in our study relapsed [26,39].

A shared limitation with the study of Schmitz et al. is the low percentage of genetically characterized cases—39% in ours and 45% in theirs. Though simplified classification may help stratify DLBCL patients prognostically, it lacks comprehensiveness. Simplified models based on classification studies with higher classification rates may offer a broader prognostic utility [15,16,17].

In addition to the high proportion of unclassified patients, the main limitation of our study is the relatively small sample size. This was a considerable obstacle in assessing the association of the studied genetic groups with the occurrence of a relapse. Schmitz’s study included a substantially larger number of patients, making a reliable comparison between our simplified approach and their more complex methodology challenging. Additionally, while Schmitz’s classification relied on both exome and transcriptome sequencing, our analysis was based exclusively on RNA sequencing. While RNA sequencing is a valid method, it may complicate cross-study comparisons [40]. However, given the complexity and ongoing evolution of prognostic molecular characterization in DLBCL, there is a growing need for complementary efforts toward developing simpler, more transparent, and widely applicable classification systems.

## 4. Materials and Methods

### 4.1. Patients

Cases with PFS < 2 years (the “Relapse” group) and those with PFS > 5 years (the “Remission” group) were identified from patients with histologically verified DLBCL, who were treated with at least one cycle of RCHOP or RCHOP-like first-line systemic therapy between 2008 and 2017 at the Institute of Oncology Ljubljana. Patients with primary CNS and testicular lymphoma were excluded. PFS was defined as time from the last cycle of the first-line systemic treatment until disease progression; OS was defined as the time from the DLBCL diagnosis until death; and lymphoma-specific OS was defined as the time from the DLBCL diagnosis until death caused by lymphoma. Clinical data of patients were collected from the institutional database and were analyzed. Data were censored in January 2025.

### 4.2. Immunohistochemistry and FISH

On diagnostic histological material, the DLBCL diagnosis and immunohistochemical COO classification were re-evaluated by a single pathologist. The material was immunohistochemically stained on Ventana Benchmark XT (Ventana Medical Systems Inc., Tucson, AZ, USA). Antibodies against CD3 (F7.2.38, Dako, Agilent Technologies, Santa Clara, CA, USA), CD5 (4C7, Ventana Medical Systems Inc., Tuscon, AZ, USA), CD10 (56C6, Novocastra, Leica Microsystems Inc., Wetzlar, Germany), CD20 (L26, Dako, Agilent Technologies, Santa Clara, CA, USA), CD21 (EP3093, CellMark, Gothenburg, Sweden), CD23 (DAK-CD23, Dako, Agilent Technologies, Santa Clara, CA, USA), Cycline D1 (DCS-6, Sigma Aldrich, Burlington, MA, USA), Ki67 (MIB-1, Dako, Agilent Technologies, Santa Clara, CA, USA), BCL2 (124, Dako, Agilent Technologies, Santa Clara, CA, USA), BCL6 (GI 191E/A8, Cell Marque, Rocklin, CA, USA), MUM1 (MUM1p, Dako, Agilent Technologies, Santa Clara, CA, USA), and MYC (9E10, Dako, Agilent Technologies, Santa Clara, CA, USA) were used.

In cases with an MYC expression > 40%, fluorescent in situ hybridization (FISH) was performed for *MYC* translocation; if positive, analyses for *BCL2* and *BCL6* translocations were also carried out. FISH was performed with ZytoLight SPEC MYC Dual Color Break Apart (8q24), ZytoLight SPEC BCL6 Dual Color Break Apart BCL6 (3q27) (ZytoVision GmbH, Bremerhaven, Germany), and Vysis LSI BCL2 Break Apart FISH Probe Kit (18q21) (Abbott, IL, USA.). A BCL2 expression > 50% was considered positive.

### 4.3. Molecular Analysis

For genetic analysis, RNA was isolated using MagMAX^TM^ FFPE DNA/RNA Ultra Kit (ThermoFisher, Waltham, MA, USA). RNA was converted into cDNA and NGS was performed using the Archer FusionPlex Lymphoma Kit (InvitaeArcherDX, San Francisco, CA, USA), which determines the expression level, point mutations, insertions, deletions, splice variants, and fusions of 125 genes that are commonly involved in lymphoma pathogenesis.

The library was quantified with the qPCR Library Quantification Kit (KAPA Biosystems, Wilmington, MA, USA), before sequencing on the MiSeqDx system (Illumina, San Diego, CA, USA). Data were analyzed using Archer Analysis version 6.0.3.2. The genetic variant was considered to be a true-positive if the allele fraction was at least 10% and the depth of coverage at the variant’s genomic position was at least 100x. Fusions were considered to be true-positive if they were covered with five or more unique reads and represented more than 10% of the reads. All reported variants were classified according to CAP/AMP/ASCO guidelines [41]. Only previously described pathogenic variants were used for the subgrouping of patients. Variants were considered pathogenic (oncogenic) if they were described in the Supplementary Table of the Schmitz’s study [14] or in the OncoKB database as oncogenic. Variants of uncertain significance that had previously not been reported in the literature or whose oncogenicity was described as inconclusive, as well as benign variants with an allele frequency in GnomAD v2.1 above 0.1%, were excluded from the analysis.

Cases were classified according to the COO classification into the ABC, GCB, and unclassified subgroups according to gene expression patterns. *MYD88^L265P^* and alterations in *CD79B*, *EZH2*, *NOTCH1*, *NOTCH2*, *BCL2*, and *BCL6* were identified and used for genetic classification. Cases with alterations in *CD79B* and *MYD88* were classified as MCD, cases with alterations in *EZH2* and/or *BCL2* were classified as EZB, cases with alterations in *BCL6* and/or *NOTCH2* were classified as BN2, and cases with alterations in *NOTCH1* were classified as N1. The remaining cases were genetically unclassified. Expression data were used as a surrogate method for detecting the amplification of the *CD79B* gene. Gene amplification was considered to be true-positive when the relative expression of the gene was above eight on the scale from zero to nine, as calculated by the Archer analysis software.

### 4.4. Statistical Analysis

Data were statistically analyzed with SPSS Statistics, version 26 (IBM, Armonk, NY, USA). Categorical variables were analyzed with the χ-square or Fischer’s exact test. Numerical variables were analyzed with the independent sample t-test or the Mann–Whitney U test if the data distribution was not normal. Multivariate analysis was performed with binary logistic or linear regression. A *p*-value < 0.05 was considered statistically significant.

## 5. Conclusions

In conclusion, our genetic groups shared certain characteristics with those identified by Schmitz et al., such as COO features and a low frequency of N1 patients. However, due to the limited sample size, definitive conclusions regarding the outcomes associated with these genetic groups cannot be drawn. Beyond the IPI, clinical features such as male sex, the presence of B symptoms, and bone marrow involvement were significantly associated with a relapse within two years following treatment. Although molecular prognostic tools are not yet routinely implemented in clinical practice, these clinical markers may help identify DLBCL patients at a higher risk of relapse.

## Figures and Tables

**Table 1:** Patient characteristics at diagnosis. DLBCL NOS—diffuse large B-cell lymphoma not otherwise specified; DLBCL/HGBCL—diffuse large B-cell lymphoma/high-grade B-cell lymphoma with *MYC* and *BCL2* rearrangements; unc—unclassified; GEP COO—gene expression profiling cell of origin; IPI—International Prognostic Index. N = 105.

Diagnosis		B Symptoms	Stage		IPI	
DLBCL NOS	102 (97%)	45 (43%)	1	16 (15%)	0–1	32 (30%)
			2	22 (21%)		
DLBCL/HGBCL	3 (3%)		3	22 (21%)	2	27 (26%)
			4	45 (43%)		
**Male sex**		**Age** (years, median)	**GEP COO** (N = 100)		3	26 (25%)
			ABC	29 (29%)		
49 (47%)		65 (range 27–89)	GCB	57 (57%)	4–5	20 (19%)
			Unc.	14 (14%)		

**Table 2:** Genetic characterization of diffuse large B-cell lymphoma cases with progression-free survival (PFS) > 5 years and those with PFS < 2 years after the first systemic treatment (the “Remission” and “Relapse” groups). unc—unclassified. N = 100.

Genetic Group	EZB	MCD	BN2	N1	Unc	Total
**“Remission” group**	10 (56%)	4 (36%)	5 (56%)	0 (0%)	33 (54%)	52
**“Relapse” group**	8 (44%)	7 (64%)	4 (44%)	1 (100%)	28 (46%)	48
**Total**	18	11	9	1	61	

**Table 3:** Clinical characteristics of patients classified into genetic groups. For numerical and categorical variables, medians and proportion (absolute number) are given, respectively. * Bone marrow involvement was evaluated in 95 genetically classified patients. ** The number of all transformed cases, including those of undetermined type indolent lymphoma origin, as well as cases transformed from follicular/marginal zone lymphoma/chronic lymphocytic leukemia, is given. IPI—International Prognostic Index; LDH—enzyme lactate dehydrogenase. N = 100.

Genetic Group	EZB (N = 18)	MCD (N = 11)	BN2 (N = 9)	N1 (N = 1)	Unc (N = 61)
**Age**	63.5	71	74	59	65
**Male sex**	33% (6)	36% (4)	56% (5)	0% (0)	52% (32)
**IPI**	3	2	2	2	2
Bone marrow involvement *	13% (2)	56% (5)	33% (3)	100% (1)	32% (19)
**Stage**	3.5	3	4	4	3
**Elevated LDH**	83% (15)	55% (6)	44% (4)	0% (0)	48% (29)
**B symptoms**	50% (9)	45% (5)	33% (3)	0% (0)	44% (27)
Indolent lymphoma transformation **	3 (2/0/0)	3 (0/1/0)	2 (0/1/0)	1 (0/0/1)	20 (7/4/2)

**Table 4:** Categorical variable comparison of diffuse large B-cell lymphoma cases with progression-free survival (PFS) > 5 years and those with PFS < 2 years after the first systemic treatment (the “Remission” and “Relapse” groups). * Bone marrow involvement was evaluated in 99 patients. *p*-values are calculated with χ-square test. GEP—gene expression profiling; WHO PS—World Health Organization performance status; ABC—activated B-cell subtype; GCB—germinal-center B-cell subtype; LDH—enzyme lactate dehydrogenase. N =105.

Clinical Characteristic	>1 Extranodal Site Involved	Elevated LDH	PS WHO > 1	Bone Marrow Involvement *	B Symptoms	Male Sex	ABC/GCB Cases (GEP)
**“Remission” group** **(N = 52)**	16 (31%)	20 (38%)	4 (8%)	7 (13%)	15 (29%)	19 (37%)	12/34
**“Relapse” group** **(N = 53)**	23 (43%)	37 (70%)	7 (13%)	25 (47%)	30 (57%)	30 (57%)	17/23
***p*-value**	0.181	0.001	0.356	<0.001	0.004	0.039	/

**Table 5:** Numerical variable comparison of diffuse large B-cell lymphoma cases with progression-free survival (PFS) > 5 years and those with PFS < 2 years after the first systemic treatment (the “Remission” and “Relapse” groups). Median values are provided. *p*-values are calculated using the Mann–Whitney U test. * MIB-1 was determined in 102 patients. IPI—International Prognostic Index. N = 105.

	IPI	Age	Stage	MIB-1 (%) *
**“Remission” group**	2	62	2	80
**“Relapse” group**	3	68	4	80
***p*** **-value**	0.001	0.356	<0.001	0.206

## Data Availability

The raw data supporting the conclusions of this article will be made available by the authors on request.

## Supplementary Materials

The following supporting information can be downloaded at: https://www.mdpi.com/article/10.3390/ijms26115087/s1.
